# Genetic Variations in the Transforming Growth Factor-β1 Pathway May Improve Predictive Power for Overall Survival in Non-small Cell Lung Cancer

**DOI:** 10.3389/fonc.2021.599719

**Published:** 2021-07-07

**Authors:** Hong Zhang, Weili Wang, Wenhu Pi, Nan Bi, Colleen DesRosiers, Fengchong Kong, Monica Cheng, Li Yang, Tim Lautenschlaeger, Shruti Jolly, Jianyue Jin, Feng-Ming (Spring) Kong

**Affiliations:** ^1^Department of Radiation Oncology, School of Medicine, University of Maryland Baltimore, Baltimore, MD, United States; ^2^Department of Radiation Oncology, Case Western Reserve University Comprehensive Cancer Center, Cleveland, OH, United States; ^3^Laboratory of Cellular and Molecular Radiation Oncology, Department of Radiation Oncology, Radiation Oncology Institue of Enze Medical Health Academy, Affiliated Taizhou Hospital of Wenzhou Medical University, Taizhou, China; ^4^Department of Radiation Oncology, National Cancer Center/National Clinical Research Center for Cancer/Cancer Hospital, Chinese Academy of Medical Sciences and Peking Union Medical College, Beijing, China; ^5^Departments of Radiation Oncology, IU Simon Cancer Center, Indiana University School of Medicine, Indianapolis, IN, United States; ^6^Michigan Medicine Radiation Oncology, University Hospital, Ann Arbor, MI, United States; ^7^Department of Clinical Oncology, The University of Hong Kong-Shenzhen Hospital, Li Ka SHing Medical School, Shenzhen, China

**Keywords:** machine learning, single nuclear polymorphism, overall survival, non-small cell lung cancer, TGF-β1

## Abstract

**Purpose:** Transforming growth factor-β1 (TGF-β1), a known immune suppressor, plays an important role in tumor progression and overall survival (OS) in many types of cancers. We hypothesized that genetic variations of single nucleotide polymorphisms (SNPs) in the TGF-β1 pathway can predict survival in patients with non-small cell lung cancer (NSCLC) after radiation therapy.

**Materials and Methods:** Fourteen functional SNPs in the TGF-β1 pathway were measured in 166 patients with NSCLC enrolled in a multi-center clinical trial. Clinical factors, including age, gender, ethnicity, smoking status, stage group, histology, Karnofsky Performance Status, equivalent dose at 2 Gy fractions (EQD2), and the use of chemotherapy, were first tested under the univariate Cox's proportional hazards model. All significant clinical predictors were combined as a group of predictors named “Clinical.” The significant SNPs under the Cox proportional hazards model were combined as a group of predictors named “SNP.” The predictive powers of models using Clinical and Clinical + SNP were compared with the cross-validation concordance index (C-index) of random forest models.

**Results:** Age, gender, stage group, smoking, histology, and EQD2 were identified as significant clinical predictors: Clinical. Among 14 SNPs, BMP2:rs235756 (HR = 0.63; 95% CI:0.42–0.93; *p* = 0.022), SMAD9:rs7333607 (HR = 2.79; 95% CI 1.22–6.41; *p* = 0.015), SMAD3:rs12102171 (HR = 0.68; 95% CI: 0.46–1.00; *p* = 0.050), and SMAD4: rs12456284 (HR = 0.63; 95% CI: 0.43–0.92; *p* = 0.016) were identified as powerful predictors of SNP. After adding SNP, the C-index of the model increased from 84.1 to 87.6% at 24 months and from 79.4 to 84.4% at 36 months.

**Conclusion:** Genetic variations in the TGF-β1 pathway have the potential to improve the prediction accuracy for OS in patients with NSCLC.

## Introduction

Lung cancer is the leading cause of cancer death and the second most commonly diagnosed type of cancer in the USA. It was estimated that 235,760 new cases would be diagnosed in 2020, accounting for about 12.5% of all cancers diagnosed, and only 23% of cases are diagnosed at an early stage (1, 2). The 5-year survival rate is only about 22.6% in the USA, though there is already a 13% improvement over the last 5 years for all lung cancers (2, 3). Approximately, 83% of patients with lung cancer are identified with non-small cell cancer (NSCLC) (4), and radiation therapy (RT) is a mainstay local treatment used for all stages of the disease (5). However, the survival benefit of RT to an individual patient varies with the baseline clinical and genetic factors of each patient. Some clinical factors, such as age, stage group, and histology, have a strong correlation with the overall survival (OS) of patients with NSCLC after RT (6). There is a need for an integrated clinical and genetic model for survival prediction.

Recent studies have shown a strong correlation between transforming growth factor- β1 (TGF-β1) and OS in various types of cancer (7). TGF-β1 is a prototype of a multifunctional cytokine and plays an important role in tumor angiogenesis, stroma formation, immune suppression, carcinogenesis, tumor metastasis progression, and prognosis for patients with cancer. Single nucleotide polymorphisms (SNPs) of TGF-β1 have been significant factors for prognosis in colon and pancreatic cancers (8, 9). We hypothesized that functional SNPs of the TGF-β1 pathway genes can regulate the TGF-β1 expression level and function of the downstream pathway genes for tumor progression and the immune system of the host, thus contributing to OS in patients with NSCLC.

## Materials and Methods

### Study Population

This study included 166 patients with inoperable stages I–III NSCLC, enrolled through prospective studies approved by the institutional review board (IRB) of participating centers. All patients signed written informed consent. Patients received definitive thoracic radiotherapy (≥55 Gy EQD2) with or without chemotherapy. All patients were treated with three-dimensional conformal RT techniques as described in previous studies (10, 11). Clinical factors, including total equivalent dose at 2 Gy fractions (EQD2), age, gender, ethnicity, smoking history, histology, stage group, Karnofsky performance score (KPS), and the use of chemotherapy, were collected prospectively.

### Selection of SNPs

We selected 14 functional SNPs present in the 11 genes responsible for the TGF-β1 pathways based on the following criteria: (1) tag SNPs in the candidate genes; (2) a minor allele frequency greater than 10%; and (3) previously reported significant findings with correlation with the outcome of RT or chemotherapy or cancer risk.

### Sample Collection and Genotyping

The buffy coat was collected from each patient before the commencement of treatment and stored at −80°C. Genomic DNA was extracted from the buffy coat using the Blood Mini Kit of Gentra® Puregene® (Qiagen, Valencia, CA) according to the protocol of the manufacturer. The concentrations of genomic DNA were measured by a Nano Drop 2000c Spectrophotometer (Nano Drop Technologies, Inc., Wilmington, DE). Quantified DNA samples were placed on a matrix-assisted laser desorption/ionization time-of-flight mass spectrometer (Sequenom, Inc., San Diego, CA) according to the protocol of the manufacturer. For pre-genotyping quality control, randomly selected samples were blindly run in duplicate or triplicate. For post-genotyping quality control, low call-rate SNPs that had a call rate of <90% in all samples or the samples that had a call rate of <90% in all SNPs were excluded from further analysis.

### Statistical Analysis

The analysis was performed with R (12), and the missing data were imputed with the most frequent values. A power analysis was performed based on the data. The Cox proportional hazards model (13) was used to carry out univariate analysis, and the random survival forest tree (14) was used to carry out multivariate analysis. For discrete clinical factors, the median survival time (MST) with 95% CIs and the 24-month survival time with 95% CIs were calculated. At first, the Cox proportional hazards model was used to estimate the hazard ratio (HR) and 95% confidence interval (CI) of each predictor. The OS and event indicator, used as the output variables, were calculated from the beginning of treatment to the last visit or death. All significant predictors (*p* < 0.05) selected from clinical factors with the univariate Cox proportional hazards model were combined as a group of predictors named “Clinical.” The independence between SNPs was tested before running a multivariate model. To show the results of the independence test, the linkage disequilibrium (LD) (15) was calculated and plotted. Then, each SNP was tested with the Cox proportional hazards model. The significant SNPs were combined as a group of predictors named “SNP.” Two models, *RModel1* and *RModel2*, were built as random survival forest trees on Clinical and Clinical + SNP, respectively. The justification for using the random survival forest tree instead of the Cox proportional hazards model as the multivariate model was given that: (1) the ensemble structure of the random survival forest tree could avoid the overfitting issue, given the limited number of patients and numerous predictors used in the study; (2) the random survival forest tree could handle both categorical and continuous predictors smoothly; (3) the Cox model assumes that continuous predictor variables have linear relationships with the risk of the event occurring, which is usually not true (16).

The predictive power of *RModel1* and *RModel2* were estimated and compared in terms of the concordance index (C-index) (17) with a 3-fold cross-validation (18). The 3-fold cross-validation randomly and evenly divided the whole data set into three groups. Then, the random survival forest classifier was trained by using two groups as training data. The trained classifier was tested using the remaining group to get the evaluation metrics. In this way, three evaluation metrics could be achieved using three disparate groups as testing data, and the mean evaluation metrics were used in the evaluation.

## Results

### Patient Clinical Factors

A total of 166 patients were included in this study. The death probability was 0.51 for the data. The postulated HR was set as 2. The postulated proportions of the sample size allotted to one group were 0.5. Type I error was 0.05, as stated above. The power of 166 patients was 0.7, which is less than the traditional 0.8, but it is still reasonable (19). The median age was 65.7 (64.1, 67.8) years. About 75.3% (68.7, 81.9%) patients received concurrent and adjuvant chemotherapy. The overall MST was 24.5 (19.3, 30.6) months, and the median follow-up time was 22.8 (9.2, 36.3) months. The clinical factors of the patients shown in Table 1, including gender (*p* = 0.0084), stage group (*p* = 0.016 for stage group 2 and *p* = 0.19 for stage group 3), smoking (*p* = 0.061 for former smokers and *p* = 0.041 for smokers), histology (*p* = 0.024 for squamous, *p* = 0.022 for large cell, and *p* = 0.0018 for other), age (*p* = 0.011), and EQD2 (*p* = 0.00024), were significant. This group of significant clinical factors was defined as Clinical. The favorable factors were female, early-stage group, no smoking, adenocarcinoma, young, and high EQD2, consistent with published studies (20). Ethnicity, the use of chemotherapy, and KPS did not show a significant correlation with survival and were not included in the multivariate analysis.

**Table 1:** Selected clinical factors of NSCLC patient population.

**Factors**	**Cases # *n* (%)**	**MST, 95% CI (month)**	**2 years survival, 95%CI (%)**	**HR (95% CI)**	***P*-value**
**Gender**					
Male	127 (76.5)	22.0	45.1 (37.2, 54.7)		
Female	39 (23.5)	38.2	65.8 (53.2, 82.7)	0.52 (0.32,0.85)	**0.0084**
**Ethnicity**					
Caucasian	158 (95.2)	24.5	50.2 (42.9, 58.7)		
No Caucasian	8 (4.8)	25.6	50.0 (25.0, 100)	0.85 (0.34,2.09)	**0.73**
**Stage**					
1	32 (19.3)	39.4	71.7 (57.7, 89.2)		
2	19 (11.4)	14.3	26.3 (12.4,55.8)	2.26 (1.16,4.38)	**0.016**
3	115 (69.3)	23.0	47.6 (39.2,57.9)	1.39 (0.85,2.27)	**0.19**
**Smoking**					
No smoking	6 (3.6)	NA	83.3 (58.3,100)		
Former smoker	79 (47.6)	23.1	48.1 (38.3,60.5)	6.64 (0.92,48.03)	**0.061**
Smoker	81 (48.8)	22.0	47.0 (36.6,60.4)	7.93 (1.09,57.55)	**0.041**
**Chemotherapy**					
No	41 (24.7)	22.0	48.7 (35.5,66.7)		
Yes	125 (75.3)	25.1	50.3 (41.7,60.6)	0.84 (0.55,1.28)	**0.43**
**Histology**					
Adenocarcinoma (1)	35 (21.1)	37.2	65.7 (51.7,83.5)		
Squamous (2)	56 (33.7)	22.2	47.5 (35.9,62.7)	1.91 (1.09, 3.34)	**0.024**
Other (3)	75 (45.2)	18.6	38.5 (27.3,54.2)	2.42 (1.41,4.17)	**0.0014**
Age				1.02 (1.005,1.04)	**0.011**
KPS				1.00 (0.98,1.008)	**0.51**
EQD2				0.97 (0.96,0.993)	**0.00024**

*MST, median survival time; HR, hazard ratio. Bold indicate statistical significance at P value of 0.05*.

The effect of clinical factors in patients with stage III NSCLC was also tested similarly, and the results were similar to that discussed above. Detailed findings were shown in the Supplementary File.

### Individual SNPs and OS

**Figure 1:**
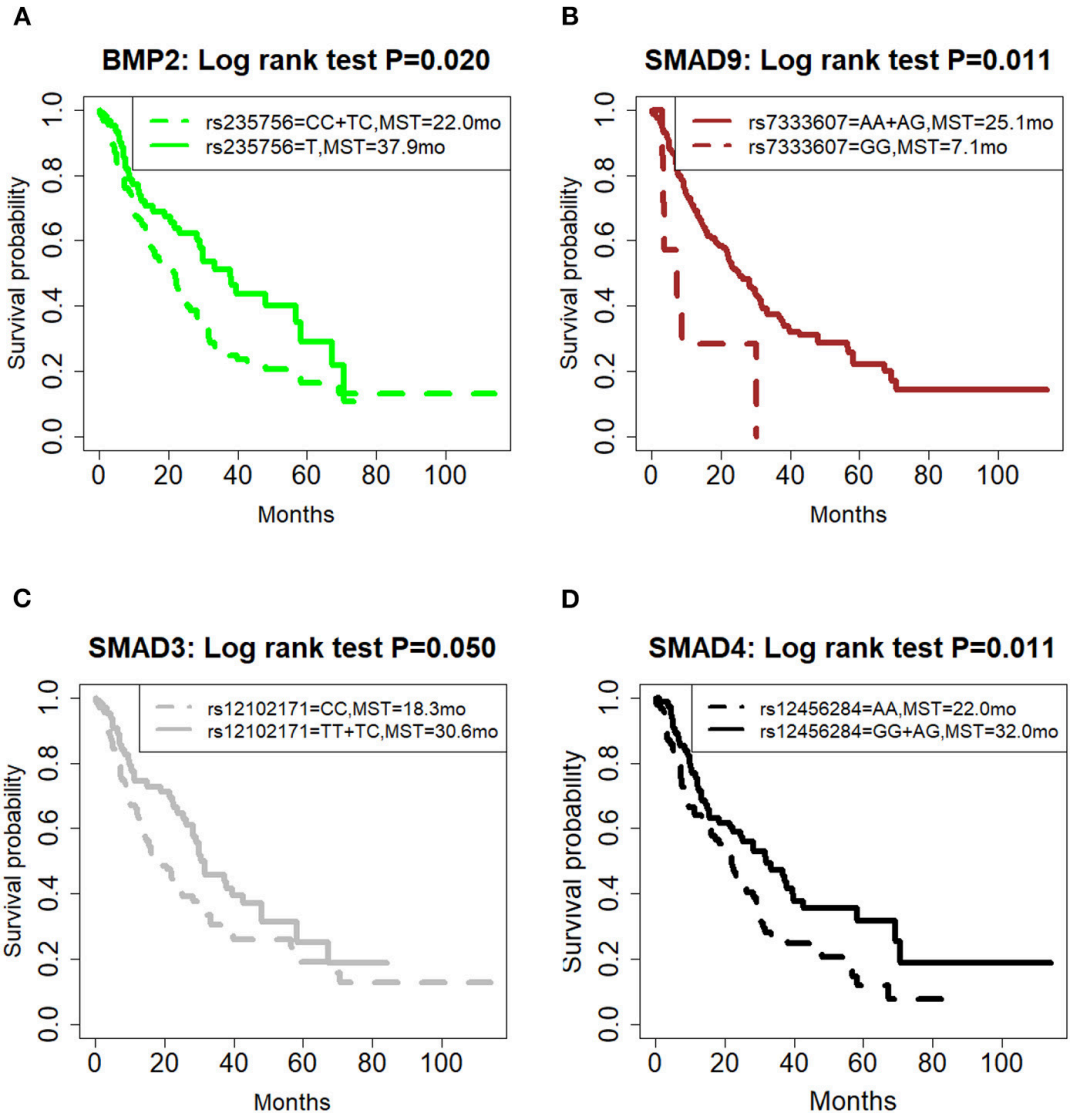
Effect of genetic variation on Kaplan-Meier overall survival curve. **(A)** BMP2:rs235756; **(B)** SMAD9:rs7333607; **(C)** SMAD3:rs12102171; **(D)** SMAD4: rs12456284; MST in months. MST, median survival time.

The correlation of all SNPs with OS was summarized in Table 2. The genetic model for each SNP followed the previous publication (21). Among them, four SNPs, including BMP2:rs235756 (*p* = 0.022), SMAD9:rs7333607 (*p* = 0.015), SMAD3:rs12102171 (*p* = 0.050), and SMAD4: rs12456284 (*p* = 0.016), were significant predictors for OS. The Kaplan-Meier (KM) plots of these four SNPs are shown in Figure 1 with *p*-values for the log-rank test listed. All *p*-values for the log-rank test were significant with the cut-off value of 0.05.

**Table 2:** Genetic correlation with OS, univariate analysis (*N* = *166*).

**Gene**	**SNP**	**Wild genotype^#^**	Model^*^	**HR for the minor allele (95%CI)**	**Effect of minor on survival**	***P*-value**
BMP2	rs235756	C (63.2%)	rec	0.63 (0.42,0.93)	**Favorable**	**0.022**
ACVR2A	rs1424954	A (34.6%)	rec	1.72 (0.92,3.18)	Unfavorable	**0.088**
BMP1	rs3857979	C (75.9%)	rec	1.17 (0.76,1.81)	Unfavorable	**0.47**
INHBC	rs4760259	C (90.7%)	rec	1.07 (0.58,2.01)	Unfavorable	**0.82**
SMAD3	rs4776342	A (58.8%)	add	0.76 (0.52,1.12)	Favorable	**0.17**
TGFB1	rs4803455	A (25.9%)	dom	1.38 (0.88,2.18)	Unfavorable	**0.16**
SMAD3	rs6494633	C (76.9%)	rec	1.06 (0.68,1.63)	Unfavorable	**0.81**
SMAD7	rs7227023	A (0.6%)	dom	1.11 (0.15,7.95)	Unfavorable	**0.92**
SMAD9	rs7333607	A (95.8%)	rec	2.79 (1.22,6.41)	**Unfavorable**	**0.015**
SMAD1	rs11724777	A (69.0%)	rec	0.76 (0.49,1.16)	Favorable	**0.20**
SMAD1	rs11939979	A (19.0%)	dom	1.02 (0.64,1.64)	Unfavorable	**0.93**
SMAD3	rs12102171	C (62.0%)	dom	0.68 (0.46,1.00)	**Favorable**	**0.050**
SMAD4	rs12456284	A (55.4%)	dom	0.63 (0.43,0.92)	**Favorable**	**0.016**
SMAD6	rs12913975	A (6.8%)	dom	1.23 (0.57,2.64)	Unfavorable	**0.60**

# *The percentage was based on our data*

* *Genetic model of inheritance: dom, dominant model; rec, recessive model; add, additive model. SNP, single nucleotide polymorphism. Bold indicate statistical significance at P value of 0.05*.

BMP2:rs235756 (HR = 0.63; 95% CI:0.42–0.93) in a recessive model showed lower risk for patients with minor allele (T). The MST increased from 22 months for patients with the wild-type (C) to 37.9 months for patients carrying the minor allele (T) (Log-rank *p* = 0.020, Figure 1A).

SMAD9:rs7333607 (HR = 2.79; 95% CI 1.22–6.41) in a recessive model was correlated with an increased risk of death among patients carrying the minor allele (G). Patients with minor allele (G) of this SNP had a significantly shorter MST of 7.1 months compared with 25.1 months for patients with the wild type (A) (Log-rank *p* = 0.011, Figure 1B).

SMAD3:rs12102171 (HR = 0.68; 95% CI: 0.46–1.00) was in a dominant model. Patients carrying the minor allele (T) had a significantly decreased risk of death. This decrease in risk resulted in an increased MST by nearly 11.8 months: from 18.8 months for those with the wild-type genotype (C) to 30.6 months for patients carrying the minor allele (T) (Log-rank *p* = 0.050, Figure 1C).

SMAD4: rs12456284 (HR = 0.63; 95% CI: 0.43–0.92) in a dominant model which correlated with a decreased risk of death among patients carrying the minor allele (G). These patients with the minor allele (G) of this SNP had a significantly longer MST of 32 months compared with 22 months for patients with the wild type (A) (Log-rank *p* = 0.011, Figure 1D).

The effect of SNPs in patients with stage III NSCLC was also tested similarly, and the results were similar to that discussed above. Detailed findings were shown in the Supplementary File.

### A Combined Model of Integrating *Clinical and SNP* Factors for Survival

**Figure 2:**
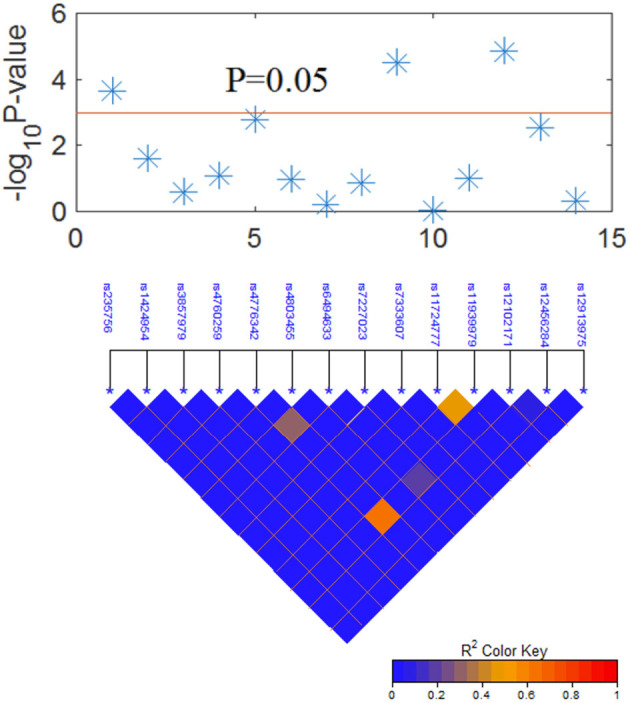
Graphical representation of the *P*-value obtained from individual SNP analysis and linkage disequilibrium (LD) structure.

The LD plot of 14 SNPs is shown in Figure 2. Most SNPs showed strong independence (*R*^2^ < 0.2). The significant SNPs were independent of each other, and the multivariate analysis of each SNP was valid.

**Figure 3:**
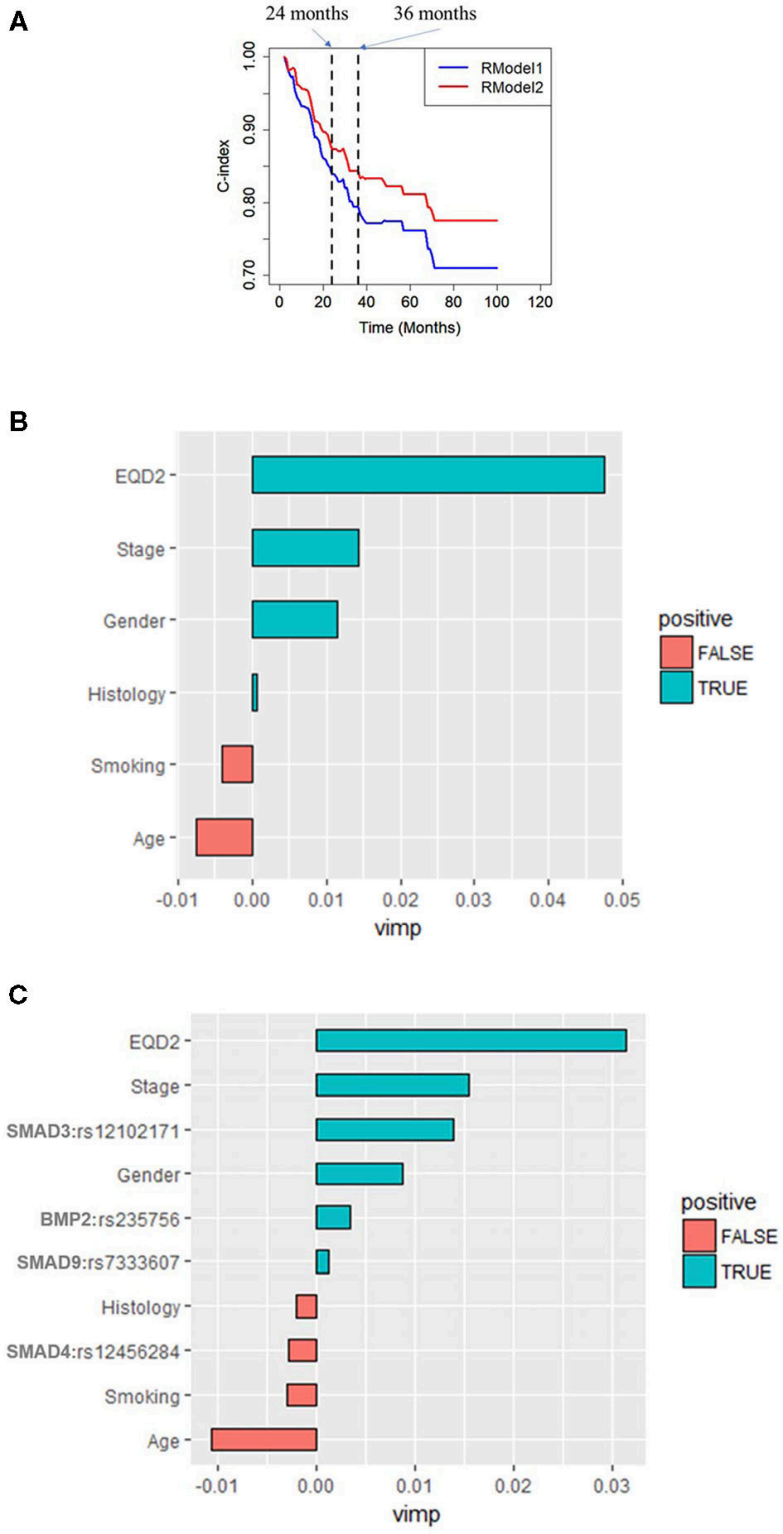
**(A)** Time-dependent C-index of *RModel1* and *RModel2*. *RModel2* increased the C-index from 0.73 to 0.78 compared with *RModel1* at 24 months. Importance of predictors (VIMP) in the random forest for **(B)** *RModel1* and **(C)** *RModel2*. *RModel1*: a model of combining only clinical predictors. *RModel2*: a model of combining significant clinical and genetic factors.

After a long-term follow-up of 18–100 months, the random forest classifier of *RModel2* with 1,000 trees trained with Clinical+SNP significantly increased the C-index compared to that of *RModel1* as shown in Figure 3A. For example, the C-index of *RModel1* at 24 months was 84.1%. After adding SNP as predictors, the C-index of *RModel2* increased to 87.6%. At 36 months, the C-index increased from 79.4 to 84.4%. A *t*-test was applied on the C-index of the two models, and the *p*-value was 0.003 for both models, which indicated that *RModel2* performed better than *RModel1* in terms of the C-index.

## Discussion

This study analyzed the correlation with clinical outcomes in patients with several adverse genotypes, and the results suggest that the cumulative influence by multiple genetic variants within the TGF-β signaling pathways could improve the prediction accuracy for survival among patients with NSCLC after RT.

The survival significance of TGF-β1 pathway genomics has a biologic rationale. The TGF-β is a prototype of a multifunctional cytokine and is the ligand for the TGF-β type I and II receptors. TGF-β composes of TGF-β1, 2, 3, and other about 30 family members, including the activin/inhibin subfamily, such as BMP subfamily (Bone Morphogenetic Proteins BMPs) and the mullerian inhibitory substance (22, 23). BMPs are the intracellular signaling members which can activate downstream signaling genes in TGF-β signaling pathways (24, 25). Smad proteins (Smad 1 through 9) are transcriptional regulators which are important for intracellular TGF-β signaling (26). In TGF-β signaling pathways, those subfamily genes have a similar effect on cell growth, cell proliferation and differentiation, and cell death and plays a key role in embryonic development, immune system regulation, and the duo roles of diseases, such as skeletal diseases, fibrosis, and cancer (23, 27–30). TGF-β signaling is very important in lung health and disease, regulating lung organogenesis and homeostasis, including alveolar cells and epithelial cells differentiation, fibroblast activation, and extracellular matrix organization. Whereas, TGF-β is the most potent epithelial-mesenchymal transition (EMT) inducer in NSCLC formation (31). DNA variants like SNPs can affect gene expressions and the functions of core disease-related genes (32).

The findings that SNPs in the TGF-β1 pathway genes can predict survival are clinically meaningful SNPs and consistent with the previous reports. Signature of TGF-β predicts metastasis-free survival in NSCLC (33, 34). SNPs of TGF-β1 gene have been reported to associate with OS in patients with NSCLC treated with definitive radio (chemo) therapy (35–37). The signature of a single SNP may only provide a modest or undetectable effect, whereas the amplified effects of combined SNPs in the same pathway may enhance predictive power (7, 38). In radiation, TGF-β1 may help in predicting radiation-induced lung toxicity (RILT) (39–41).

The SNPs identified in the study with prognostic values are consistent with reports from other investigators on their significance in other cancers (42–44). BMP2:rs235756 is in the downstream region of the BMP2 gene and has already been shown to alter normal BMP function. Several studies suggested that BMP2:rs235756 increased the production of the BMP protein and the concentration of serum ferritin levels, which promoted BMP signaling in cancer progression (42–44). BMP2 is highly expressed in lung cancer and is involved in regulating lung cancer angiogenesis and metastasis (45, 46). Silencing the expression of BMP-2 inhibits lung cancer cell proliferation and migration (47). BMP2:rs235756 has previously been reported as a significant biomarker for OS in patients with lung cancer (21). For patients who underwent RT, BMP2:rs235756 was shown to predict radiation pneumonitis (48), which is an important clinical outcome.

Furthermore, this study also suggested that SMAD3:rs12102171 correlated with OS in NSCLC. SMAD3:rs12102171, located in the intron region between exon3 and 4 of the SMAD3 gene, is known for its function as a mediator of TGF-β pro-fibrotic activities. Inflammatory cells and fibroblasts without smad3 do not auto-induce TGF-β, but Smad3 null mice are resistant to radiation-induced fibrosis (49). TGF-β/Smad3 signaling plays critical roles in biological processes, such as epithelial-mesenchymal transition (EMT) lung cancer cell progression and lung cancer patient survival (21, 50). That report showed a significant correlation with osteoarthritis (51). SMAD9:rs7333607 is located in the intron region of the SMAD9 gene and only correlated with lung cancer survival (21).

Smad4 belongs to the Smad gene family, acts as a mediator of TGF-β signaling pathways (26), and was classified as a tumor suppressor gene which plays important roles in maintaining tissue homeostasis and suppressing tumorigenesis (1). The loss of SMAD4 expression significantly correlated with poor OS in patients with cancers, such as pancreatic cancer, colorectal cancer, and prostate cancer (52, 53). The SNP rs12456284 locates 3′ UTR region of the Smad4 gene, was predicted to influence the potential miRNA binding, and downregulate the gene expression with Smad4 associated with gastric cancer (54). Genetic variants in the BMP/Smad4/Hamp hepcidin-regulating pathway, such as Hamp rs1882694, BMP2 rs1979855, rs3178250, and rs1980499, were associated with OS, local-regional progression-free survival, progression-free survival, and distant metastasis-free survival in patients receiving definitive RT for NSCLC but not rs12456284 (55).

In a tree analysis of the study, the variable importance (VIMP) measures the increase (or decrease) in prediction error for the forest classifier when a variable is randomly “noised-up.” A large positive VIMP shows that the prediction accuracy of the forest classifier is significantly degraded when a variable is noised-up. Thus, a large VIMP shows a more predictive variable. The VIMP of each variable in the *RModel1* and *RModel2* are listed in Figures 3B,C. It is shown that EQD2 and stage group were always two important predictors in the two models. SMAD3:rs12102171 was more important than other predictors, except for EQD2 and stage group, which was not reported before. BMP2:rs235756 and SMAD4: rs12456284 have a similar importance as smoking, which has been consistently shown as an important predictor in the clinical OS of patients with NSCLC. SMAD9:rs7333607 was less important and it may be overlooked should the results be validated by independent studies.

The present study has several limitations. First, this study has limited statistical power because of the small sample size in each stage group and the analysis of the limited number of SNPs. Second, the selection of the SNPs was rather arbitrary, which was limited by the published data at the start of this study. Additional SNPs candidates may be further identified; future studies can use the methodology of the study to develop better models with the inclusion of more candidates and more external validations. Although it showed the promise of genetic variation in guiding personalized medicine, the study shall be considered exploratory. The findings should be validated by an independent study population.

## Conclusions

In this study, we systematically evaluated genetic variations in the TGF-β1 pathway as predictors of the outcomes for patients with NSCLC treated with RT. Four SNPs (SMAD3:rs12102171, BMP2:rs235756, SMAD9:rs7333607, and SMAD4: rs12456284) showed strong correlations with OS in patients with NSCLC after RT. The current model improves prediction accuracy by adding genetic variations in the TGF-β1 pathway.

## Data Availability Statement

The datasets presented in this study can be found in online repositories. The names of the repository/repositories and accession number(s) can be found below: NCBI (accession numbers are: SCV001478478–SCV001478481).

## Ethics Statement

The studies involving human participants were reviewed and approved by IRB. The patients/participants provided their written informed consent to participate in this study. Written informed consent was obtained from the individual(s) for the publication of any potentially identifiable images or data included in this article.

## Author Contributions

Concept and design: JJ, HZ, and F-MK. Acquisition, analysis, or interpretation of data: HZ, WW, WP, NB, JJ, and F-MK. Drafting of the manuscript: HZ, JJ, and F-MK. Critical revision of the manuscript for important intellectual content: WW, NB, WP, FK, MC, LY, TL, and SJ. Statistical analysis: HZ, CD, and JJ. Obtained funding: F-MK. Administrative, technical, or material support: WW, WP, and NB. Study supervision: JJ and F-MK.

## Conflict of Interest

The authors declare that the research was conducted in the absence of any commercial or financial relationships that could be construed as a potential conflict of interest.
