# Supplementary material for: Genetic Variations in the Transforming Growth Factor-β1 Pathway May Improve Predictive Power for Overall Survival in Non-small Cell Lung Cancer
Source: Front Oncol. 2021 Jul 7;11:599719. doi: 10.3389/fonc.2021.599719 (PMC8294034; doi:10.3389/fonc.2021.599719)
Supplement: Supplementary file 1 [file Data_Sheet_1.pdf]

Based on 96 stage 3 patients with chemotherapy

**Factors**	**HR (95%CI)**	**P-value**
Gender		
Male		
Female	0.45 (0.25,0.86)	**0.014**
Ethnicity		
Caucasian		
No Caucasian	1.2 (0.30,5.02)	0.78
Smoking		
No smoking		
Former smoker	253 (0,Inf)	0.99
Smoker	277 (0,Inf)	0.99
Histology		
Adenocarcinoma (1)		
Squamous (2)	2.41 (1.14, 5.08)	**0.021**
Other (3)	2.96 (1.47,5.99)	**0.0025**
Age	1.02 (0.99,1.04)	0.114
KPS	1.00 (0.98,1.009)	0.54
EQD2	0.95 (0.94,0.99)	**0.0014**

HR: hazard ratio.

**SNPs**	**HR for the minor allele (95%CI)**	**P-value**
rs235756	0.63 (0.37,1.07)	0.087
rs1424954	1.06 (0.47,2.38)	0.89
rs3857979	0.89 (0.50,1.59)	0.70
rs4760259	0.94 (0.45, 1.97)	0.87
rs4776342	0.55 (0.34,0.91)	**0.020**
rs4803455	1.77 (0.97,3.23)	0.06
rs6494633	0.95 (0.53,1.70)	0.86
rs7227023	1.16 (0.16,8.40)	0.88
rs7333607	10.9 (3.10,38.47)	**0.00020**
rs11724777	0.93 (0.54,1.62)	0.80
rs11939979	1.01 (0.54,1.89)	0.97
rs12102171	0.45 (0.27,0.74)	**0.0018**
rs12456284	0.61 (0.38,0.98)	**0.041**
rs12913975	0.76 (0.35,1.67)	0.50
rs235756	2.1 (0.76,5.82)	0.15

HR: hazard ratio.
